# Mitochondria and Energetic Depression in Cell Pathophysiology

**DOI:** 10.3390/ijms10052252

**Published:** 2009-05-19

**Authors:** Enn Seppet, Marju Gruno, Ants Peetsalu, Zemfira Gizatullina, Huu Phuc Nguyen, Stefan Vielhaber, Manfred H.P. Wussling, Sonata Trumbeckaite, Odeta Arandarcikaite, Doreen Jerzembeck, Maria Sonnabend, Katharina Jegorov, Stephan Zierz, Frank Striggow, Frank N. Gellerich

**Affiliations:** 1 Department of Pathophysiology, University of Tartu, Tartu, Estonia; E-Mail: marju.gruno@ut.ee (M.G.); 2 Department of Surgery, University of Tartu, Tartu, Estonia; E-Mail: ants.peetsalu@ut.ee (A.P.); 3 KeyNeurotek AG, ZENIT-Technology Park Magdeburg, Magdeburg, Germany; E-Mails: zemfira.gizatullina@rambler.ru (Z.G.); Doreen.Jerzembek@gmx.de (D.J.); Msmaria1987@aol.com (M.S.); jegorov@gmx.de (K.J.); frank.striggow@keyneurotek.de (F.S.); frank.gellerich@keyneurotek.de (F.N.G.); 4 Department of Medical Genetics, University of Tübingen, Tübingen, Germany; E-Mail: hoa.nguyen@med.uni-tuebingen.de (H.P.N.); 5 Department of Neurology, Otto von Guericke University, Magdeburg, Germany; E-Mail: stefan.vielhaber@med.ovgu.de (S.V.); 6 Bernstein Institute for Physiology, Martin-Luther-University Halle-Wittenberg, Germany; E-Mail: manfred.wussling@medizin.uni-halle.de (M.H.P.W.); 7 Institute for Biomedical Research, Kaunas University of Medicine, Kaunas, Lithuania; E-Mails: sonatai@centras.lt (S.T.); arandarcikaite@yahoo.com (O.A.); 8 Department of Neurology, Martin-Luther-University Halle-Wittenberg, Germany; E-Mail: stephan.zierz@medizin.uni-halle.de (S.Z.)

**Keywords:** mitochondria, energy depression, mitochondrial cell death, neurodegenerative diseases, inflammation, hypoxia, cancer

## Abstract

Mitochondrial dysfunction is a hallmark of almost all diseases. Acquired or inherited mutations of the mitochondrial genome DNA may give rise to mitochondrial diseases. Another class of disorders, in which mitochondrial impairments are initiated by extramitochondrial factors, includes neurodegenerative diseases and syndromes resulting from typical pathological processes, such as hypoxia/ischemia, inflammation, intoxications, and carcinogenesis. Both classes of diseases lead to cellular energetic depression (CED), which is characterized by decreased cytosolic phosphorylation potential that suppresses the cell’s ability to do work and control the intracellular Ca^2+^ homeostasis and its redox state. If progressing, CED leads to cell death, whose type is linked to the functional status of the mitochondria. In the case of limited deterioration, when some amounts of ATP can still be generated due to oxidative phosphorylation (OXPHOS), mitochondria launch the apoptotic cell death program by release of cytochrome c. Following pronounced CED, cytoplasmic ATP levels fall below the thresholds required for processing the ATP-dependent apoptotic cascade and the cell dies from necrosis. Both types of death can be grouped together as a mitochondrial cell death (MCD). However, there exist multiple adaptive reactions aimed at protecting cells against CED. In this context, a metabolic shift characterized by suppression of OXPHOS combined with activation of aerobic glycolysis as the main pathway for ATP synthesis (Warburg effect) is of central importance. Whereas this type of adaptation is sufficiently effective to avoid CED and to control the cellular redox state, thereby ensuring the cell survival, it also favors the avoidance of apoptotic cell death. This scenario may underlie uncontrolled cellular proliferation and growth, eventually resulting in carcinogenesis.

## 1. Introduction

The importance of impaired mitochondrial function in cellular pathophysiology was first recognized by Otto Warburg, who proposed that development of cancer is causally related to an altered energy metabolism due to suppression of OXPHOS and activation of glycolysis [1]. Later it was shown that clinically expressed hypermetabolic state also resulted from dysfunction of mitochondria [2] and that defects in mitochondrial metabolism due to mutations in mitochondrial DNA (mtDNA) and nuclear DNA (nDNA) underlie various encephalomyopathic syndromes (Table 1) [3–6]. Based on these studies, the concept of mitochondrial diseases [7] was introduced, which considers these diseases to be manifested in many different tissues, but primarily caused by defects in OXPHOS due to mutations of mitochondrial proteins (Table 1).

The concept of mitochondrial medicine was successful in promoting a massive search for new DNA mutations underlying the pathogenesis of different diseases (see http://www.mitomap.org and recent reviews [8–11]). At the same time, genetic approaches have suffered from serious limitations in disclosing the underlying pathomechanisms.

Firstly, they do not provide an explanation for enormous phenotypic variety of clinical manifestations of mitochondrial dysfunction [12], partially because in most cases disorders of mitochondria either are not primarily causal for the disease, or their disease-specific role has not been revealed [13–26]. Secondly, the underlying genetic defects are still unknown for about 50% of the adult patients and for 80 – 90% of sick children [10].

Thirdly, the gene-based concepts of disease largely ignore the complex physiological properties of mitochondria (impermeable membranes and transmembrane gradients for many compounds [27], specific osmotic behavior, and the fission – fusion equilibrium [28]) that underlie a large network of relations between mitochondria and cells. Different alterations in these networks may lead to variable phenotypic presentations of the disease states.

Fourthly, gene-function relationships can not be fully assessed because the function of some 160 out of 1,200 mitochondrial proteins is still unknown [29].

**Figure 1.**
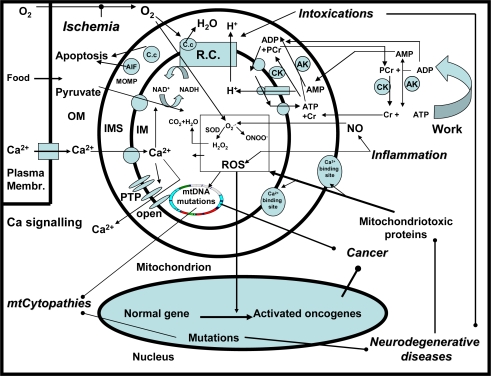
The central role of mitochondria in mitochondrial diseases, neurodegenerative diseases, inflammation, ischemia, intoxication and cancer.

For these reasons and in the light of the recent knowledge, the subsequent discussion provides arguments in favour of idea that the earlier gene-based paradigm of mitochondrial diseases has to be changed into a more general concept which considers that mitochondrial disorders play a central role in majority of pathological processes largely due to their critical function in controlling the cellular energy status, signaling systems and the pathways of cell death. To illustrate this, the Figure 1 depicts the main functions of mitochondria and shows how different clinical entities result in disease-specific impairments of these functions. Most importantly, mitochondria produce ATP for cell work. The mechanism responsible for that process is based on coupling of oxidation of reducing equivalents (e.g. NADH) and electron transport along the respiratory chain to synthesis of ATP by F_0_F_1_ATPase, driven by the proton gradient regenerated by the action of the respiratory chain. OXPHOS is coupled to phosphocreatine (PCr) shuttle, which adjusts high phosphorylation potentials for cell work and transports PCr (the ATP equivalent) to the ATP-utilizing enzymes and Cr (the ADP equivalent) back into the intermembrane space (IMS).

In this system the mitochondrial and extramitochondrial creatine kinases (CK) work in opposite directions, and are functionally coupled to adenine nucleotide translocase (ANT) and ATPases, respectively. Similarly, extramitochondrial adenylate kinase (AK) converts ADP into ATP and AMP and contributes to the ATP regeneration. AMP diffuses as ADP equivalent into the IMS where mitochondrial AK converts AMP and ATP to ADP. In the case of failure or lack of CK-mediated energy transfer pathway, the cytosolic ADP may largely increase in response to increased activity of ATPases, and ADP may diffuse directly into the (IMS) to stimulate OXPHOS. In ischemia the mitochondrial inner membrane (IM) becomes permeable due to the opening of the permeability transition (PT) pore and the outer membrane (OM) becomes leaky, which results in release of the apoptosis inducing factor (AIF) and cytochrome c. These changes induce apoptosis or necrosis, depending on the cellular levels of ATP. In parallel, reactive oxygen species (ROS) are formed due to retarded flow of electrons in the respiratory chain. Normally, O_2_^−.^ can be eliminated by mitochondrial Mn-dependent superoxide dismutase (MnSOD), a component of the antioxidant defence system. When the ROS formation exceeds the defence capacity, dangerous ROS attack on all biomolecules occurs (oxidative stress), acutely reducing the activity of respiratory chain enzymes, but chronically impairing the mitochondrial and nuclear DNA. In inflammation, increased NO production reinforces the oxidative stress in mitochondria via the reversible inhibition of the respiratory chain and formation of peroxynitrite (ONOO^−^) from O_2_^−.^ and NO [30,31–39]. Chronic ROS is assumed to cause gene mutations responsible for cancerogenesis. Hereditary mutations in the mitochondrial genome cause the mitochondrial cytopathies (mt-cytopathies), e.g. due to impairment of respiratory chain complexes. In neurodegenerative diseases, hereditary mutations in non-mitochondrial genes cause the formation of cytotoxic proteins, which give rise to mitochondrial dysfunction. Mitochondriotoxic actions of these pathological proteins are realized by interactions with regulatory Ca^2+^- binding sites localized at the surface of mitochondria [40]. Ca^2+^ ions can be accumulated by mitochondria e.g. via the uniporter. Excessive Ca^2+^ in the matrix can induce the opening of the PT pore (PTP). In case of reversible PT the mitochondria release a fraction of Ca^2+^ that serves as a signaling messenger, but in conditions of irreversible PT the mitochondria deteriorate and die that leads to serious pathophysiological consequences [41,42]. Finally, the intoxication of mitochondria by medicaments or by specific toxins often impairs the respiratory chain and can cause acute impairments and in some cases symptomatic neurodegenerative diseases.

## 2. The Cellular Energetic Depression and Mitochondrial Cell Death as Cornerstones of the Diseases

**Figure 2.**
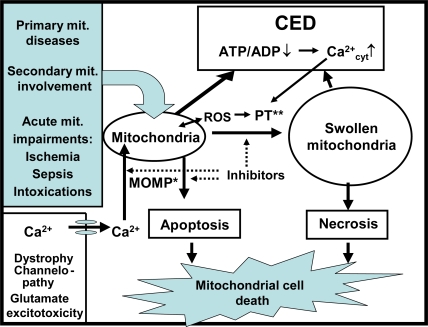
Mitochondrial cell death. Loss of mitochondrial capacity to synthesize ATP in the processes of OXPHOS leads to cellular energetic depression (CED) characterized by decreased cytosolic phosphorylation potential and increased cytosolic Ca^2+^ concentration (Ca^2+^cyt) that leads to reduced ability of cell to do work. The resulting ROS formation and Ca^2+^ overload further impair the structure and function of mitochondria. In mild stage of CED, when mitochondria can generate some amounts of ATP, mitochondria launch a program of apoptotic cell death by release of cytochrome c. At pronounced CED, when the cytoplasmic ATP levels fall below the levels required for processing the ATP-dependent apoptotic reactions, the cell dies from necrosis. Both, the apoptotic and necrotic death pathways that are mediated by mitochondrial impairments can be classified as of mitochondrial cell death (MCD). The molecular mechanism of mitochondrial outer membrane permeabilization (MOMP) and PT are potential targets for therapeutic interventions preventing mitochondrial cell death.

There exist a number of different cell death programs, such as the apoptotic, autophagic, cytoplasmic, and other types [43], initiated and progressing in the course of neurodegenerative, infectious, traumatic, ischemic, and metabolic diseases. For all these diseases and death types, mitochondrial involvement is a common phenomenon, because the pathological processes such as inflammation, hypoxia/ischemia, intoxications, metabolic blockade, and oxidative stress exert deleterious effects on structure and function of mitochondria (Figure 1, Table 2) [43–45]. As an outcome, a decline in cytosolic phosphorylation potential ensues that inhibits the function of Ca^2+^-ATPases, thereby causing cytosolic Ca^2+^ overload and suppression of the cell’s ability to work (Figure 2).

This condition, for which we propose the term cellular energetic depression (CED), is characterized by a mismatch between ATP production and utilization and enforces mitochondria to accumulate large amounts of Ca^2+^ that induces the permeability transition (PT) of the mitochondrial inner membrane [46]. This process includes mitochondrial swelling, collapse of the membrane potential (ΔΨ), and splitting of ATP due to reversal of mitochondrial F_0_F_1_ATPase. If mitochondria shift into irreversible PT, the cell must die by necrosis. The CED pathway can also start due to intracellular Ca^2+^ overload caused by degenerative changes of the cell membrane, or channelopathies that lead to increased Ca^2+^ flux into the cell [47]. Whereas all of these disturbances result in cell’s death, the functional status of mitochondria plays a key role in determining which type of MCD will prevail. If the pathogenic signals induce mitochondrial outer membrane permeabilization (MOMP) [43,44] and CED is relatively mild (i.e. OXPHOS is not yet markedly compromised), cytochrome c and the AIF are released to initiate and execute the apoptotic machinery [43]) (Figure 2). However, along with progression of CED, cellular ATP level may become lower than required for supporting apoptosis with the consequence that cells will enter the necrotic pathway of death. In this review, we provide ample evidence for the involvement of mitochondrial disturbances not only in the regulation of cell death, but also in the progression of major pathological processes, such as inflammation, hypoxia, and carcinogenesis (see Sections 3.2. – 3.4) that all are causal for cell death. Considering the central role of mitochondria in pathogenetic processes, we propose that all forms of cell death, including apoptotic and necrotic cell death, as well as all other death pathways that are linked to mitochondrial impairments, should be envisaged as MCD. MCD contributes to degeneration or atrophy of the affected tissue, and can occur slowly, as in the case of neurodegenerative diseases, or rapidly, as in rhabdomyolysis. Correspondingly, the mitochondria should be regarded as key targets for pharmacological treatment, in order to protect cells from MCD. Indeed, there exist several potent inhibitors of PT, such as cyclosporin A (CsA) [43] and sanglifehrin A [48], that make it possible to attenuate or cure the atrophic processes. It has been shown that CsA can increase the lifespan and decrease the symptoms of degeneration in collagen VI – deficient mice [49]. Also, MOMP, realized via a cascade of molecular reactions should be inhibitable [43,44]. To provide more effective approaches in pharmacological interventions, it may be necessary to discriminate between cell death pathways where mitochondria participate and those that occur without mitochondrial involvement [43]. To our knowledge, the term MCD was first proposed by Kroemer *et al.*, but was only used for the mitochondrial apoptotic pathway including the MOMP [50], a newly discovered phenomenon at that time. We propose to use the term MCD for all modes of death where irreversible mitochondrial impairments, irrespectively of the primary reason and underlying mechanism, are involved.

Studies in the last decades have resulted in understanding that in many types of cells, especially those characterized with high energy turnover, the cellular energy metabolism represents a strictly organized system in which mitochondria and ATPases are linked to each other by specialized energy transfer pathways formed by isoenzymes of creatine kinase (CK) and adenylate kinase (AK) (Figure 1) [51–55]. Therefore, CED (Figure 2) is related not only to injuries of mitochondria, but also to alterations of the energy transfer system. For example, the CK-phosphotransfer network is known to be compromised in conditions of cardiac diseases, e.g. in heart failure [56–58]. The underlying mechanisms include reduced myocardial creatine levels [57], downregulated expression of mitochondrial CK (mi-CK) [57,58], and decoupling between the mi-CK and adenine nucleotide translocase (ANT) [59], these changes suppressing mitochondrial synthesis of phosphocreatine (PCr). On the other hand, reduced expression of extramitochondrial CK isoforms and their inhibition by AMP kinase [58,60,61] decrease the effectiveness of ADP rephosphorylation near ATPases at the expense of PCr [57]. Impaired interaction between mi-CK and ANT might favor opening of the mitochondrial PT pore, thus leading to CED and MCD [62–64]. Fortunately, dysfunction of the CK-phosphotransfer system can be more or less compensated by activation of the AK-mediated system of energy transfer [65,66], because the mitochondrial AK isoform is functionally coupled to ANT and accumulation of ADP near ATP-utilizing enzymes favors using its β-phosphoryls by AK for ATP formation [65]. Also, an inefficient CK system can to some extent overcome by direct ATP/ADP diffusion in order to ensure energy transfer and feedback [67,68].

In the case of neurodegenerative diseases [e.g. Parkinson’s disease (PD), Alzheimer’s disease (AD), Huntington’s disease (HD), and amyotrophic lateral sclerosis (ALS)] mitochondria and the energy transfer systems may be impaired by toxic and disease-specific proteins. Mutated huntingtin binds to the MOM, suppresses the mitochondrial capacity of Ca^2+^ accumulation [69], and hinders the axonal mobility of mitochondria [70]. In normal brain cells, the function of HK II depends on its interaction with the MOM and the cytoskeletal network [71,72]. On the other hand, mi-CK, localizing in the intermembrane space, is functionally coupled to ANT [73]. Both of these mitochondrial kinases participate in the formation of the PT pore [74,75] and both of them suppress mitochondrial ROS production, due to their stimulative effect on OXPHOS [76,77]. In the case of AD, the CK activity is suppressed, owing to the structural alterations in the brain cell’s cytoskeleton [78]. Decreased CK activity in the spinal cord was also observed in G93A transgenic mice model of familiar ALS [79]. Thus, the neurodegenerative diseases may be associated with impaired functional coupling of mitochondrial kinases to OXPHOS, which could contribute to CED and ROS [80,81].

Recent data demonstrate impairment of CK-mediated energy transfer pathway in sepsis, as the mi-CK activity was found to be reduced and its coupling to OXPHOS impaired in the diaphragm and heart of endotoxin-treated dogs [82].

### 2.1. Role of cytosolic Ca^2+^ in regulation of mitochondrial life and death

**Figure 3.**
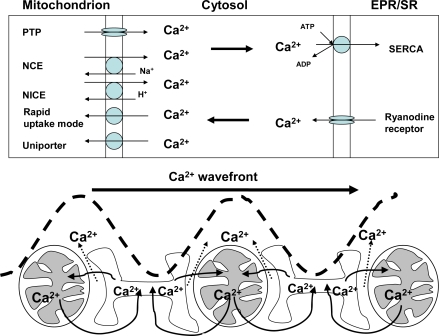
Involvement of mitochondria and endoplasmic/sarcoplasmic reticulum (EPR/SR) in Ca^2+^ signaling. Upper panel: Mitochondria accumulate Ca^2+^ via the uniporter and by the rapid uptake mode. Accumulated Ca^2+^ can be released from mitochondria through reversible Na^+^ independent (NICE) or Na^+^ dependent pathways (NCE), but the rates of Ca^2+^ efflux via these pathways are low in comparison to the fast Ca^2+^ efflux via the PT pore that can be opened reversibly or irreversibly [46]. Ca^2+^ accumulation by EPR/SR is realized by SERCA which requires ATP at sufficiently high phosphorylation potentials. To avoid inhibition of SERCA by increasing ADP, it is rephosphorylated by the PCr shuttle (Figure 1). Lower panel: Mitochondria and EPR/SR interact in order to control cytosolic Ca^2+^ waves and their directed propagation, as modeled in the reconstituted gel system (Table 3 [86]).

It has been known for a long time that [Ca^2+^]_cyt_ exerts a regulatory impact on OXPHOS [83,84] and that Ca^2+^ overload impairs the mitochondrial function [85]. As shown in Figure 3, Ca^2+^ enters the matrix through the uniporter located in the inner membrane. This process is driven by ΔΨ and can be inhibited by ruthenium red. The K_M_ of the uniporter in brain and muscle mitochondria is about 3 μM Ca^2+^ [40], which is higher than the cytosolic Ca^2+^ concentration ([Ca^2+^]_cyt_) that usually fluctuates between 50 nM and 1 μM [87]. In the light of these data, substantial Ca^2+^ uptake into mitochondria can not be expected. However, [Ca^2+^]_cyt_ is dynamically compartmentalized and spatio-temporally changing, so that its local concentration can reach the values over 40 μM even at normal cellular circumstances [88]. Moreover, polyamines such as spermine, which exist at millimolar concentrations in the cytosol, markedly decrease the K_M_ for Ca^2+^ uptake by isolated mitochondria [89,90]. Under these conditions, mitochondria can take up large amounts of Ca^2+^ via uniporter, supported by formation of appatite [91]. On the other hand, Ca^2+^ can be released from the mitochondrion via the Na^+^ independent (NICE) and/or the Na^+^ dependent (NCE) exchange pathways, and via the fast Ca^2+^ efflux aided by the PT pore. The capacity of the latter exceeds that of NICE and NCE [92] (Figure 3).

The phenomenon of [Ca^2+^]_cyt_-induced stimulation of mitochondrial respiration [83,84] has been attributed to accumulation of Ca^2+^ in the matrix and activation of mitochondrial enzymes, such as pyruvate dehydrogenase (PDH), isocitrate dehydrogenase, and 2-oxoglutarate dehydrogenase [83]. However, due to low sensitivity of dehydrogenases to Ca^2+^ (S_0.5_ 0.8 – 5 μM [83]), these enzymes likely can not contribute to activation of OXPHOS at lower (nanomolar) ranges of [Ca^2+^]_cyt_. Furthermore, increases in Vmax of state 3 respiration due to activation of dehydrogenases are small [83] and computer models assuming the intramitochondrial Ca^2+^ regulation of OXPHOS can not simulate the measured data [93]. These arguments question the predominant role of intramitochondrial dehydrogenase-mediated mechanisms in regulation of OXPHOS by Ca^2+^.

**Figure 4.**
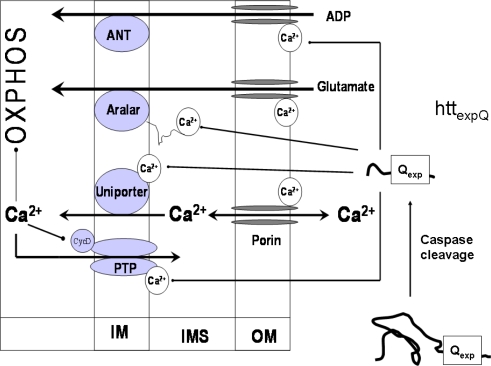
Mechanisms of regulation of OXPHOS by [Ca^2+^]_cyt_ that stimulates mitochondrial respiration and ATP synthesis by binding to regulatory sites of several proteins in the mitochondrial outer compartment [40], such as the porin pore [96,98], the PT pore [99], the Ca^2+^ uniporter, and aralar [94,95]. The Ca^2+^ binding sites of transporters, PT pore and VDAC may also represent the targets for various pathogenic proteins. As discussed in chapter 3.1.1, huntingtin with an expanded poly Q tract (htt_expQ_) cleaved by caspases [100,101] can interact with the regulatory Ca^2+^ binding sites of PT pore and transporters, thereby disturbing the regulation of OXPHOS by [Ca^2+^]_cyt_ that causes energetic depression, mitochondrial cell death, and tissue atrophy [40].

Recent data show that aralar 1 [94,95] and citrin, which are the brain and liver isoforms of the Ca^2+^-dependent aspartate/glutamate carrier, respectively, contain regulatory Ca^2+^ binding sites localized in the intermembrane space that enable the regulation of both carriers by [Ca^2+^]_cyt_ (Figure 4). Remarkably, aralar 1 is activated at much lower Ca^2+^ concentrations (S_0.5_ = 0.3 μM, [94]) than required for activation of the Ca^2+^ uniporter. It is a component of the malate/aspartate shuttle that transports the reducing hydrogen into mitochondria [94]. Alternatively, glutamate can enter the mitochondrial matrix by the glutamate/H^+^ symporter, but the activity of that carrier is low in most organs, except liver and kidney [96–97]. Pardo *et al.* have demonstrated that elevation of [Ca^2+^] increases hydrogen transport into brain mitochondria [94], and Palmieri *et al.* have detected a Ca^2+^- activation of the glutamate/aspartate carrier, by registrating increased rates of mitochondrial glutamate decarboxylation in human cell line HET-293T in response to enhancement in [Ca^2+^] [95]. Moreover, other mitochondrial substrate carriers possess the regulatory Ca^2+^ binding sites to sense [Ca^2+^]_cyt_ as well. Among them, the Ca^2+^-regulated ATP-Mg/P_i_ carrier [102–105] belongs to a subfamily of human Ca^2+^ binding mitochondrial carriers, named as short Ca^2+^ binding mitochondrial carriers [105]. Three of them are isoenzymes of the ATP-Mg/Pi carrier, responsible for the net flux of adenine nucleotides into or out of mitochondria. Ca^2+^ binding motives in the N-terminus of these carriers may serve as sensors of [Ca^2+^]_cyt_ [103]. Notably, because the mitochondrial Ca^2+^-uniporter exposes a regulatory Ca^2+^ binding site into the intermembrane space, it can be activated by extramitochondrial Ca^2+^ [106,107]. It has been also shown that the PT pore has external binding site for divalent cations, and occupation of that site by Ca^2+^ and Mg^2+^ is expected to decrease the PT pore open probability [99]. Finally, the porin pore of the mitochondrial outer membrane, termed as voltage-dependent anion channel (VDAC), is regulated by [Ca^2+^]_cyt_ [98,108]. VDAC is responsible for the passage of mitochondrial metabolites with a molecular weight < 1,000 Da, but it seems also to participate in formation of the PT pore. Increases in extramitochondrial Ca^2+^ markedly enhance the permeability of VDAC, probably, through the effect on glutamine residue in position 72 of VDAC, a regulatory Ca^2+^ binding site of that protein [108].

Our recent data show that the complex I dependent state 3 respiration with glutamate/malate is much lower than complex II dependent respiration with succinate in brain mitochondria if the incubation medium contains very low amounts of Ca^2+^ [40]. However, the respiration of mitochondria strongly increases in response to elevated [Ca^2+^] (S_0.5_ = 0.35 μM). This effect is also observed in the presence of ruthenium red, a blocker of Ca^2+^ uniporter, which means that activation is exclusively mediated by extramitochondrial Ca^2+^ [40]. Considering these novel data, the role of interaction of Ca^2+^ with mitochondrial functions needs to be re-estimated. Firstly, we propose that [Ca^2+^]_cyt_ exerts significant control over the OXPHOS, independently of entering the mitochondrial matrix (Figure 4). Secondly, it seems reasonable to assume that reversible mitochondrial Ca^2+^ accumulation, well characterized in many experiments (Figure 3), is not as much required for stimulation of OXPHOS than for fulfilling other tasks. For example, mitochondrial Ca^2+^ retention may be crucially involved within the redistribution of [Ca^2+^]_cyt_, to avoid harmful effects while accumulating in very high concentrations in the close proximity of mitochondria and Ca^2+^ channels of the cell membrane or EPR/SR [109–111]. In addition, mitochondrial Ca^2+^ cycling may be vital for governing the cytosolic Ca^2+^-waves [86,112,113]. To address the latter function of mitochondria within the the intracellular Ca^2+^ homeostasis, we assessed the spontaneous Ca^2+^ waves by confocal scanning fluorescence microscopy, using fluo-3 as a Ca^2+^ sensor in a cell free system (an agarose gel supplemented with isolated SR vesicles, cardiac mitochondria, CK-based ATP regenerating system, and 5 mM ATP) [86]. We found that in such system lacking mitochondria, Ca^2+^ waves propagated along the gel (Figure 3) with a wavefront velocity of 40 μm/s (Table 3). When the Ca^2+^-ATPase (SERCA) was inhibited with 10 nM thapsigargin, the velocity of Ca^2+^ wave propagation decreased by 50% compared to the controls (Table 3).

In the presence of mitochondria, the velocity of the wavefront spreading strongly exceeded the velocity measured in the absence of mitochondria, and after addition of antimycin A, an inhibitor of complex III of respiratory chain, a significant retardation of the wave spreading down to the values measured in the absence of mitochondria, was determined (Table 3). These experiments led to the conclusion that mitochondria exert their control of intracellular Ca^2+^ signaling via two modes of regulation, (i) through providing sufficient ATP for Ca^2+^-ATPases in the plasmamembrane and EPR/SR and (ii) due to their ability to accumulate and release Ca^2+^ [86,112,113] (Figure 3). However, in the case of CED, reduced availability of mitochondrial ATP would suppress the activity of Ca^2+^ ATPases, which in turn would induce a rise in [Ca^2+^]_cyt_ and affect Ca^2+^-mediated signaling. This assumption was confirmed by studies on fibroblasts isolated from patients with Leigh disease, which revealed that the Ca^2+^ uptake by the EPR was significantly reduced due to energy deficiency [114].

## 3. Involvement of Mitochondria in Pathological Processes and Diseases

### 3.1. Mitochondria in neurodegenerative diseases

Abnormal protein aggregation and/or inclusion body formation are common factors underlying cellular and molecular mechanisms for neurodegenerative diseases such as AD, PD, HD, ALS, and prion diseases [115–118]. Despite a large clinical and pathophysiological heterogeneity the neurodegenerative diseases have a common ground – the pathophysiologal involvement of mitochondrial impairments [118]. In this review, the energetic aspects of HD and PD are addressed.

#### 3.1.1. Huntington’s disease

HD is a progressive neurodegenerative disorder caused by a CAG repeat expansion in the coding region of the huntingtin (htt) gene resulting in an expanded polyglutamine stretch in the huntingtin (htt) protein (httexpQ) [119,120]. The CAG repeat length of httexpQ correlates inversely with the time point of disease onset [121]. Unmodified htt itself and httexpQ are abundantly expressed in most tissues [120]. To date, numerous proteins have been detected that interact under *in vitro* conditions with htt [117], but neither the biological function of htt nor the mechanism of cytotoxic action of httexpQ is understood [117].

The symptoms of HD are motor abnormalities including chorea and psychiatric disturbances with gradual dementia, and autopsies have revealed atrophic changes in the striatum [122]. The HD patients also lose body weight despite normal or above-average food intake [123–125], which suggests impairment of energy metabolism. Indeed, alterations in energy metabolism, such as elevated lactate and malonate, decreased activities of the respiratory chain complexes, and abnormal mitochondrial morphology has been found in different brain structures of HD patients [126–132].

Accumulating evidence shows important role of altered mitochondrial Ca^2+^ signaling in the pathophysiology of HD. Indeed, decreased Ca^2+^ accumulation capacities of mitochondria isolated from brain of YAC72Q mice [133], liver of htt111Q mice [134], HD patient’s lymphocytes [133], and htt111Q striatal progenitor cells [135] have been reported. Impaired mitochondrial function and Ca^2+^ dyshomeostasis were also detected in PC12 cells after transfection with httexpQ plasmids [136]. In contrast, increased Ca^2+^-loading capacities were observed in HD brain mitochondria from several HD mice lines [137,138].

**Figure 5.**
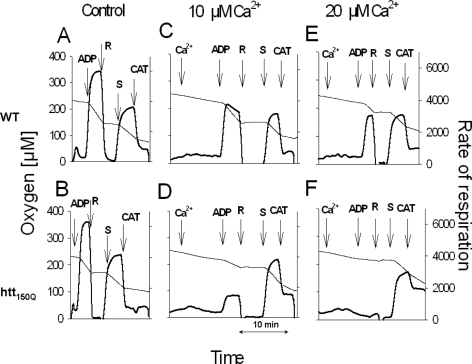
Ca^2+^-induced inhibition of pyruvate-dependent respiration in isolated muscle mitochondria of transgenic R6/2 HD mice. Multi-substrate inhibitor titration of respiration of isolated mitochondria from skeletal muscle of wild-type (WT) (A,C,E) and transgenic mice (htt_150Q_) (B,D,F) at the age of 14 to 16 weeks. Isolated muscle mitochondria (0.5 mg/mL) were incubated with 10 mM pyruvate and 2 mM malate. Additions: 10 or 20 μM Ca^2+^ as indicated; ADP, 2 mM ADP; R, 20 μM rotenone; S, 10 mM succinate; CAT, 10 μM CAT. Thin lines indicate the oxygen concentration in the oxygraph (left ordinate) whereas thick lines represent the rate of respiration in nmol O_2_/min/mg mitochondrial protein (right ordinate). The height of peaks correlates with the rate of respiration. State 3_pyr_ respiration was adjusted by addition of ADP. Rotenone, an inhibitor of complex I, completely inhibited this respiration. Subsequently, succinate addition allowed the measurement of state 3_suc_ respiration. Due to addition of carboxyatractyloside (CAT), the adenine nucleotide translocator (ANT) was inhibited and the state 4 could be measured. Note, that the pyruvate peak (state 3_pyr_) is absent in the presence of 20 μM Ca^2+^ in HD mitochondria. Further details see [139].

Recently, we presented the first detection of impaired OXPHOS phosphorylation in HD mitochondria from skeletal muscle of R6/2 mice (139). Isolated mitochondria were investigated respirometrically as described previously (140). As shown in Figure 5, exposure of htt150Q mitochondria to elevated Ca^2+^ concentrations caused a pronounced inhibition of complex I dependent respiration (Figure 5D,F) compared to wild-type (Figure 5C,E).

In general, the succinate, complex II dependent respiration was much less affected, regardless whether htt150Q or WT mitochondria were used. Furthemore, we identified a compromised mitochondrial function in fibroblasts from a HD patient with htt43Q [141]. *In situ* measurements of mitochondrial respiration revealed a specific decline of OXPHOS in HD150Q striatal cells following NMDA receptor-induced Ca^2+^ stress [138]. A Ca^2+^-induced decrease of respiration was also observed in mitochondria isolated from htt111Q striatal cells [135]. Obviously, dysfunction of HD mitochondria and increased extramitochondrial Ca^2+^ concentration are linked to each other. Therefore, some authors assumed that HD mitochondria are secondarily impaired by elevated Ca^2+^ [142]. In contrast, we hypothesized that httexpQ directly elicits the mitochondrioxic properties. To prove this hypothesis, we developed an experimental protocol allowing the investigation of OXPHOS under conditions of increasing extramitochondrial Ca^2+^ concentration in EGTA medium [40]. Experiments were performed using transgenic 21- to 27-months-old HD rat strain with 51 glutamate repeats (htt51Q). In contrast to the htt150Q R6/2 mice, a model of juvenile form of HD [143], this htt51Q rat strain exhibits specifically an adult-related onset of the neurological HD phenotype [144]. We found that the mitochondria from brain of HD rats with htt51Q showed a deficient state 3 respiration, a lower sensitivity to Ca^2+^ activation and a higher susceptibility to Ca^2+^-dependent inhibition. Furthermore, htt51Q mitochondria exhibited a diminished membrane potential stability in response to increased Ca^2+^, and lower capacities and rates of Ca^2+^ accumulation [40].

Since a mitochondrial localization of htt and httexpQ has been detected [133,136,145], direct interaction of httexpQ with proteins in the mitochondrial outer compartment can be envisaged (Figure 4). The glutamate aspartate carrier aralar [94,95] may be a candidate for one of such httexpQ interacting proteins. This carrier provides a regulatory Ca^2+^ binding site, which is exposed into the mitochondrial inner membrane space where it is activated by extramitochondrial Ca^2+^ in the nanomolar range [94]. Therefore, we consider aralar as important target of HD-related effects of httexpQ [40]. Conceivably, extended polyglutamine stretches of httexpQ interact with the regulatory Ca^2+^ binding site localized in the N-terminal sequence of aralar. As a result, Ca^2+^-dependent activation of aralar could be affected, leading to insufficient substrate supply for the mitochondrial respiratory chain. We assume that httexpQ could also interact with other Ca^2+^ binding sites localized at the mitochondrial surface as mentioned above (Figure 4). Besides aralar, other potential target proteins of httexpQ, such as the external Me^2+^ binding site of the PT pore, deserve attention [40,99]. Indeed, we have provided first evidence for interaction of htt51Q with a regulatory Ca^2+^ binding site of the PT pore, by revealing that htt51Q effects are opposite on aralar and PT [40]. Due to such interaction, aralar becomes deactivated, followed by a limited mitochondrial substrate supply and thus, a decreased activation of respiration by extramitochondrial Ca^2+^ concentrations ≤ 2 μM. In parallel, htt51Q sensitizes the PT pore to extramitochondrial Ca^2+^ levels > 1 μM, leading to its opening and suppression of respiration even in the presence of RR. It is therefore conceivable that htt51Q interacts with the Ca^2+^ binding site of the PT pore, thereby blocking the protective effect of extramitochondrial Ca^2+^ against PT (Figure 4). This effect could explain the increased susceptibility of htt51Q mitochondria to PT and their compromised Ca^2+^ retention capacity [40]. Other potential targets of httexpQ are the isoenzymes of the ATP-Mg/Pi transporters [102,104,105], the assumed Ca^2+^ binding site of the porin pore [108], and the regulatory Ca^2+^ binding site of the Ca^2+^-uniporter [70] that, after interacting with httexp, could cause a decrease in mitochondrial Ca^2+^ accumulation [40].

While considering the impact of httexpQ protein on mitochondria, it remains to be clarified as to whether this protein can indeed penetrate through the outer membrane of mitochondria in order to reach and interact with the regulatory Ca^2+^ binding sites of aralar. It is known that proteolytic degradation products of httexpQ protein are more toxic than the intact protein [100]. Furthermore, truncated httexpQ with a size of 3 and 16 kD causes htt-specific protein aggregates along with their cytotoxicity [101]. On the other hand, the dynamic rearrangements of mitochondrial structure can facilitate trapping of cytosolic proteins, including mutated htt. This can occur during fusion/fission-dependent changes, or in a course of formation of contact sites between the mitochondrial outer and inner membranes [146,147]. As these contact sites represent large protein aggregates [146] characterized by rapid redistribution of lipid components [146], they may be able to bind and translocate toxic proteins into the intermembrane space. Based on these arguments, we propose that mutated htt and its truncated polypeptides interact with Ca^2+^ binding sites in the outer compartment of mitochondria, thereby being responsible for dysregulation of mitochondrial function, CED, MCD, and tissue atrophy in HD [40].

#### 3.1.2. Parkinson’s disease

The involvement of mitochondrial dysfunction in pathogenesis of PD was suggested by the discovery that 1-methyl-4 phenyl-1,2,3,6 tetrahydropyridine (MPTP) causes parkinsonian syndromes by acting through inhibition of complex I of the respiratory chain [148] observed in the substatia nigra [149,150] and platelets of PD patients [151]. In skeletal muscle biopsies of PD patients, the activities of different complexes of the respiratory chain were found to be reduced, in association with increased flux control coefficients of complex I and IV and increased number of point mutations in mtDNA [152].

The etiology of the mitochondrial dysfunction in PD is still unclear. However, this dysfunction can be elicited by MPTP [153], rotenone [154], paraquat [155], endogenous ROS [156], and isoquinolines [157]. So far, mutations or polymorphisms in mtDNA [158,159] and at least in nine nuclear genes were identified as cause or risk factors for PD. The mutated proteins are α-synuclein, the ubiquitin E3 ligase parkin, the antioxidant protein DJ-1, the tensin homologue (PTEN)-induced kinase 1 (PINK1), the leucine-rich-repeat kinase (LRRK2) and the serine protease HTRA2, which are directly or indirectly connected to mitochondrial function [160–170].

α-Synuclein is a major component of the Lewy bodies and its mutations are associated with increased formation of oligomeric and fibrillar aggregates which promote abnormal protein accumulation or degradation with oxidative stress and mitochondrial dysfunction. Overexpression of α-synuclein in transgenic mice impairs mitochondrial function, increases oxidative stress and enhances the MPTP-induced pathology of the substantia nigra [160]. Moreover, overexpression of the A53T mutant α-synuclein gene causes a direct damage of mitochondria [161]. In contrast, an α-synuclein knock-out mice were resistant against MPTP and mitochondrial toxins, e.g., malonate and 3-nitropropionic acid [162].

Mutations in parkin and DJ-1 are associated with autosomal recessive juvenile PD. Parkin-knockout *Drosophila* [163] and mice [164] strains exhibit impaired mitochondrial function and increased oxidative stress. Leucocytes from patients with parkin mutations showed decreased complex I activities [165]. It is known that parkin can associate with the MOM and thereby prevent mitochondria against swelling and cytochrome c release, but these protective effects are abolished after mutations in parkin protein [166]. The function of DJ-1 protein seems to be the protection of cells against oxidative stress, as it can act as a redox sensor of oxidative stress that causes its translocation into mitochondria. The C106 mutation of DJ-1 prevents this translocation and induces mitochondrial dysfunction [167]. DJ-1 knock-out results in a normal mice phenotype, but sensitizes the animals to toxicity of MPTP, as seen from loss of dopaminergic neurons in response to MPTP [168].

PINK1 is a kinase localized in mitochondria, and it is also considered to be involved in neuroprotection. Overexpression of wild-type PINK1 prevents apoptosis under basal and stauroporine-induced conditions by hindering cytochrome c release, whereas mutated PINK1 antagonizes this effect [169]. PINK1 deficient *Drosophila* exhibits increased sensitivity to the complex I inhibitor rotenone [170].

It is largely accepted that degeneration of dopaminergic neurons in PD is associated with microglial-mediated inflammation and neurotoxicity (reviewed by Hald and Lotharius [171] and also below). Activation of inflammation is suggested by the finding that PD patients and animal models of PD that were treated with lipopolysaccharide (LPS), MPTP, rotenone or 6-hydroxydopamine exhibited elevated antibody levels against proteins modified by dopamine oxidation products, increased concentrations of cytokines (IL-1, IL-6, IL-10 and TNF-α), and augmented ROS production (171). All these changes were associated with impaired function of complex I of the respiratory chain in dopaminergic neurons. It is likely that modifications of biomolecules by ROS and dopamine-quinones trigger microglia activation that in turn will further promote neurotoxicity [171].

### 3.2. Mitochondria as mediators and targets of inflammation

Inflammation associates with and complicates many pathological conditions, e.g. cardiac ischemia and reperfusion, cardiac failure, neurodegenerative diseases, diabetes mellitus, and cell necrosis. Increased production of ROS is a hallmark of inflammation [172,173] and recent evidence suggests the mitochondria to be a primary source of ROS generation. This was demonstrated in experiments using mice with targeted disruption of the UCP-2 gene, which exhibited the activation of macrophage phagocytosis and ROS production, in association with increased expression of inducible NO synthase (iNOS), augmented NO production, increased resistance to NO-induced apoptosis, a greater expression of inflammatory cytokines (interferon-γ (IFN-γ) and tumor necrosis factor-α (TNF-α), faster nuclear translocation of nuclear factor-κB (NF-κB), and elevated migration ability compared to wild-type mice in response to bacterial LPS challenge [174,175]. Because TNF-α inhibits mitochondrial oxidation of NADH and FADH2-linked substrates, in association with inhibition of the respiratory chain complexes, it also increases ROS [176–178]. ROS in turn stimulates the expression of proinflammatory cytokines, such as interleukin – 2 (IL-2), TNF-α, and IL-10 [179,180], and activates NF-κB, a common target for TNF-α and IL-1 [181]. While seeking for the mechanisms linking the mitochondrial effects of TNF-α to activation of NF-κB, Itoh *et al.* [178] found that Dok-4, one of the downstream of tyrosine kinase (Dok) proteins, recruits the cytosolic c-Src protein kinase to be translocated into mitochondria and causes its activation, these changes leading to suppression of complex I and increased mitochondrial ROS production. Mitochondrial ROS and mobilization of Ca^2+^ trigger the following signalling system comprising of a cascade of kinases (TAK1, MEKK1, NIK, and IκB kinase (IKK)) that eventually activate the NF-κB [182]. Notably, the mitochondria are also the source and target of reactive nitrogen species (RNS), since IL-1, TNF-α and ROS stimulate expression of the iNOS and mitochondrial NOS-l isoforms [30,174,175,183]. NO, while accumulating in relatively low concentrations, reversibly inhibits respiration at the level of cytochrome oxidase (COX) by competing with O_2_; it also inhibits the activity of the complex II and oxidizes ubiquinol [31, 32]. At higher concentrations, it reacts with superoxide thereby forming a strong oxidant, peroxynitrite (ONOO^−^). Through S-nitrosylation and/or nitration ONOO^−^ inhibits irreversibly many mitochondrial proteins including the subunits of the complex I and II of the respiratory chain [30] which results in suppression of OXPHOS but increase in ROS production [33–39]. Very recently, a correlation between the electron flux level through the respiratory chain and the type of the mechanism/degree of NO inhibition of respiration, depending on availability in the cell of cytochrome c at the COX site, has been observed in solution and intact lymphocytes [39].

Under conditions of sepsis the endotoxin-induced impairment of mitochondrial function in heart and skeletal muscles manifests as decreased state 3 respiration caused by diminished activities of the complexes I + III as well as II + III [184,185]. Probably, these effects are related to differential effects of TNF-α on isoenzymes of NOS, because TNF-α upregulates iNOS, but downregulates eNOS [176,186–189]. The TNF-α-mediated downregulation of eNOS causes an inhibition of mitochondrial biogenesis, which is positively controlled by eNOS [190]. Suppression of mitochondrial biogenesis in conjunction with the inhibitory effects of NO and ONOO^−^ on mitochondrial respiratory chain should strongly decrease the OXPHOS capacity of the cells. Moreover, in conditions of associated sepsis, CED may worsen, due to inhibition of mitochondrial CK, as demonstrated in the diafragm and heart of endotoxin-treated dogs [82]. In the case that pathogenic bacteria are involved, the mode of action of cytokines on mitochondria is strongly augmented, because bacteria stimulate mitochondrial ROS production via direct effects on mitochondrial membranes. For example, *Helicobacter pylori* (*H. pylori*), a major pathogen causing inflammation of gastric mucosa in humans (see also Section 3.4.1), permeabilizes the MOM through translocation of the N-terminal 34 kDa fragment of *H. pylori* vacA cytotoxin into the mitochondria [191]. This process is associated with depolarization and fragmentation of mitochondrial membranes in association with suppressed ATP synthesis [192,193] and increased production of ROS, NO and ammonia, all of which secondarily exert cyto- and mitochondriotoxic effects [194–196].

Normally, ROS produced by mitochondria are largely detoxified by mitochondrial Mn-dependent superoxide dismutase (MnSOD). However, under conditions of NO excess, this enzyme undergoes nitration that inhibits its own activity [197] and, due to inactivation of NADP^+^-dependent isocitrate dehydrogenase by ONOO^−^, less glutathione (GSH) will be regenerated [198]. These cascades facilitate inflammation through establishing the feed-forward circles, as inflammation increases the mitochondrial ROS and RNS and the latter compounds again promote expression of proinflammatory cytokines.

That the cells recruit mitochondria to mediate inflammation seems to be surprising, particularly if one considers that cellular energy metabolism is largely shifted from OXPHOS towards glycolysis under inflammation conditions [172,199]. This could in turn suggest a decreased importance of mitochondria. Nevertheless, the existing evidence indicates that even under conditions of a decreased number of mitochondria, cell fate is maintainly controlled by the remaining ones. On the one hand, mitochondria mediate the prosurvival mechanisms in inflammatory cells. For example, different types of cytokines (e.g. IL-3, IL-5) suppress proapoptotic changes, such as translocation of Bax to the mitochondria, cytochrome c release, activation of caspases, and caspase-independent loss of ΔΨ, as seen in neutrophils and eosinophils [200,201]. NO, a product of inflammatory reactions, also inhibits apoptosis, via suppression of caspases (*S*-nitration) and PT pore opening, but stimulation of antiapoptotic Bcl2 [200]. In addition, mitochondria sensitize the inflammatory cells to necrotic death, thereby aggravating the inflammatory tissue lesions and complicating the disease phenotype. In this regard, it has been shown that peripheral blood lymphocytes (T cells) of patients with systemic lupus erythematosus displayed persistent mitochondrial hyperpolarization associated with increased ROS production and cellular ATP and GSH depletion, leading to necrotic death in response to IL-10, in contrast to the cells of healthy patients which exhibited transient increase in the ΔΨ that was linked to apoptotic death [201–203]. Along with this evidence, some data suggest that intact mitochondrial function is necessary for supporting the anti-inflammatory properties of the neutrophils, irrespectively of its role in controlling the apoptotic processes [204].

Interestingly, the ways how mitochondria influence the inflammatory processes vary depending on the type of the inflammatory cell and its mode of activation. For example, during differentiation of esosinophils mitochondria loose their capacity to respire and produce ATP, but retain their ability to generate ΔΨ at the expense of glycolytic ATP and to induce apoptosis via cytochrome c release [205]. By these characteristics, eosinophils differ from other inflammatory cells, e.g. neutrophils and macrophages, in which mitochondria contribute to inflammation not only by regulating apoptosis, but also by production of ATP, ROS, and RNS. On the other hand, a remarkable finding is that the Th1 or Th2 cytokines are causal for qualitatively different responses in macrophages. The Th1-derived cytokines (e.g. IFN-γ, TNF-α, LPS, and IL-1) activate the classical signalling pathways predominantly via HIF-1α leading to activation of glycolysis, increased production of NO, ROS, and proinflammatory cytokines (e.g. TNF-α, IL-1, IL-6, and IL-12), which cause marked tissue damage by amplifying the inflammatory reactions [172,199,206]. In contrast, the Th2 cytokines (e.g. IL-4 and IL-13) stimulate oxidative mechanisms via activation of STAT6 (signal transducer and activator of transcription 6) and PGC-1β (PPARγ-coactivator-1β), which induce macrophage programs for fatty acid oxidation and mitochondrial biogenesis. So, it appears that oxidative metabolism is strongly required for establishing the anti-inflammatory phenotype, which helps to limit inflammation and promote reparative processes, such as wound healing and granuloma formation, via secretion of chitinases, chemokines, and collagen [206,207]. In support of this assumption, it has been shown that recovery from *Staphylococcous aureus*-induced sepsis in mouse liver was associated with activation of mitochondrial biogenesis [208]. This process was clearly controlled by the time-dependent activation of Akt, PKC, and PKA [208]: On day first, activation of PI3K/Akt system, which promoted prosurvival through induction of antiapoptotic reactions, stimulation of mitochondrial biogenesis by phosphorylation of NRF-1, and stabilization of interaction of HK with mitochondria (as shown in Section 3.3), was observed. On day second, activation of PKC-ɛ started, which exerted protection against PTP opening and apoptosis [209,210]. Then, on a third day, activation of PKA was detected which could (i) increase mitochondrial respiratory activity through phosphorylation of complex I subunit [211,212] and (ii) suppress apoptosis by phosphorylation of BAD [213]. As a result, these mechanisms were able to restore oxidative metabolism as an early and important prosurvival factor in liver cells [208].

### 3.3. Energy metabolism in hypoxia

Tissue hypoxia resulting from oxygen supply-demand mismatch can develop in exercising skeletal muscle, especially in high altitude-hypoxic environment [214] and in conditions of tissue hypoperfusion, as in ischemic myocardium or in the core part of solid tumors [215]. In any of these circumstances, mitochondria represent both sensors and targets of hypoxia. It has been proposed that the electron transport chain reacts to hypoxia as an O_2_ sensor, by releasing ROS due to retarded flow of electrons along the respiratory chain that reduces the cytochromes and increases a lifetime of the ubisemiquinone radical in complex III [216]. Mitochondrial ROS activate the hypoxia-inducible transcription factor 1α (HIF-1α) [216–223], probably via its stabilization mediated by p38 mitogen-activated protein kinase (MAPK) [221] or by inhibition of HIF prolyl hydroxylases (HPHs) due to oxidation of ferrous iron in the catalytic domain [224]. HIF-1α is a potent inducer of gene transcription [all genes encoding glycolytic enzymes, glucose transporters, vascular endothelial growth factor (VEGF), erythropoietin, and insulin-like growth factor (IGF-2)] [222,223], which enables the cells to survive during the hypoxia period. In parallel, HIF-1α induces pyruvate dehydrogenase kinase 1 (PDK1), which suppresses the mitochondrial O_2_ consumption by phosphorylating of E1α subunit of PDH [225,226]. Under hypoxic conditions, HIF-1α also regulates cytochrome oxidase subunits by increasing the expression of LON, a mitochondrial protease that degrades COX4-1 and in parallel, activates the expression of COX4-2. This COX4-1 to COX4-2 transition optimizes the efficiency of OXPHOS through increasing the COX activity, respiration rate, and ATP production, while ROS production under conditions of reduced oxygen availability in mitochondria is suppressed [227].

Due to a shift from OXPHOS to glycolysis a new balance between the cellular energy and redox states is achieved. Therein, ATP is predominantly produced by glycolysis whereas NAD^+^, necessary for ATP production, is regenerated by LDH. Furthemore, the mitochondrial ROS production is reduced. Neumann *et al.* [228] have shown that constitutive stabilization of HIF-1α in murine thymocytes leads to overexpression of SERCA2 and diminished intracellular Ca^2+^ transients in response to T cell receptor stimulation [228]. On the contrary, HIF-1α null cardiac myocytes exhibit suppressed activity of SERCA2 [229]. Thus, HIF-1α not only determines the balance between OXPHOS and glycolysis, but also helps to avoid excess of intracellular Ca^2+^accumulation, thereby favoring maintenance of cell’s viability.

Energy depletion due to hypoxia or ischemia exerts a direct metabolic regulation through changes in the cellular adenine nucleotides, as an increased cellular AMP/ATP ratio activates the AMP-activated protein kinase (AMPK) [230]. Activation of AMPK is known to stimulate fatty acid uptake and oxidation in muscle cells, through inhibition of acetyl-CoA carboxylase due to its phosphorylation, which in turn suppresses the malonyl-CoA levels, thereby increasing the uptake of long-chain acyl-CoA to mitochondria [230]. The influence of AMPK on oxidative metabolism is also mediated by its stimulatory effects on mitochondrial biogenesis, as it increases the activity of transcription factor NRF1 and expression of co-activator PGC-1α [230,231]. On the other hand, AMPK increases the rate of glycolysis by upregulating the glucose uptake and activities of glycolytic enzymes [230]. In parallel to these effects, AMPK promotes apoptotic cell death, via phosphorylation of IRS-1 that leads to inhibition of phosphatidylinositol 3-kinase/Akt (PKB/Akt) signalling [232], and via promoting translocation of proapoptotic proteins (e.g. Bax) into mitochondria, mediated by activation of p38 MAPK downstream of AMPK [233]. Collectively, these changes, being directed to maximally support the ATP synthesis in conditions of limited availability of oxygen, serve as the cellular adaptive reactions to hypoxia.

### 3.4. Role of mitochondria in carcinogenesis

**Figure 6.**
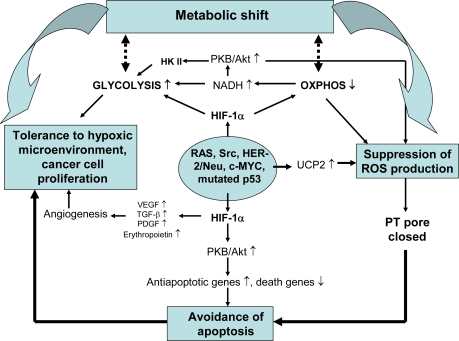
Mechanisms of mitochondrial involvement in cancer development. Activation of oncogenes and HIF-1α, a typical feature of cancer cell, is associated with downregulation of OXPHOS, its coupling to glycolysis via HKII and upregulation of UCP2 which suppress generation of ROS in mitochondria. This change together with altered balance between anti- and pro-apoptotic genes at mitochondrial membranes decreases the susceptibility of cells to apoptotic death. On the other hand, proliferation and survival of cancer cells is promoted by glycolysis and angiogenesis, both activated by HIF-1α.

The metabolic shift from OXPHOS to aerobic glycolysis (Warburg effect), tolerance to hypoxic microenvironment, ability to control ROS levels and avoidance of apoptosis are the hallmarks of cancer cells, greatly contributing to their viability, autonomous growth, migration and chemoresistance [234–242] (Figure 6).

#### 3.4.1. Mechanisms of metabolic shift in cancer cells

To date, multiple mechanisms of control over the balance between mitochondrial and glycolytic systems have been disclosed. The metabolic shift is primarily driven by specific oncogenes such as RAS, Src, HER-2/Neu, c-MYC, and p53 that activate in response to diverse stresses [243–247]. Activation of c-MYC upregulates the LDH-A isoform [245,247], whereas activation of RAS, Src, and HER-2/Neu triggers induction of glycolytic enzymes through stabilization of HIF-1α. On the other hand, both HIF-1α and c-MYC induce the expression of PDK1, that, by decreasing the activity of pyruvate dehydrogenase (PDH), downregulates the OXPHOS (see above) [248]. Suppression of OXPHOS and activation of glycolysis are feed-forward processes, as they further promote upregulation of HIF-1α through accumulation of pyruvate, other glycolytic intermediates, and oxaloacetate, all of which cause inactivation of the HIF-1α PHDs. As a result, von Hippel-Lindau protein dissociates from HIF-1α, thereby blocking its proteosome-dependent degradation, which increases the levels of active HIF-1α even in aerobic conditions [249–251]. Similarly, in conditions when succinate dehydrogenase is diminished, accumulation of succinate in the cytoplasm hinders the activity of HIF-1α PHDs that stabilizes HIF-1α at high level of activity [252–255]. All these HIF-1α mediated mechanisms strongly promote carcinogenesis in different cell types.

Besides the mechanisms based on activation of HIF-1α, there exist other mechanisms responsible for promoting the metabolic shift. Among those, p53-mediated pathways are of importance [246,256,257]. Normally, activation of p53 leads to stimulation of OXPHOS and mitochondrial respiration, because it stimulates expression of Synthesis of Cytochrome c Oxidase 2 (SCO2) protein that is necessary for the assembly of COX complex [246]. At the same time, p53 suppresses the activity of phosphoglyceromutase and glucose phosphate isomerase and brakes down the PKB/Akt mediated expression of glycolytic enzymes, thus acting as a negative regulator of glycolysis [256,257]. The function of p53 in coordinating OXPHOS and glycolysis is mediated through its interaction with AMPK pathways [258,259]. For example, under glucose deprivation, AMPK causes activation of p53 that results in cell cycle arrest and ensures survival until restoration of glucose supply does occur [258]. At the same time, AMPK can not be activated in the cells lacking p53 [259]. In cancer cells the functions of p53 described are more or less lost due to the mutations in that oncogene. Among many consequences to that change, coordination between OXPHOS and glycolysis [256,257] might be impaired, due to which the OXPHOS would be suppressed, but glycolysis upregulated, similarly to that what would be expected under the influence of HIF-1α (Figure 6).

A balance between glycolysis and OXPHOS can be controlled by the changes in common metabolites as well [245,260]. Because LDH competes with mitochondria for NADH participating in mitochondrial NADH/NAD^+^ shuttle systems [261], the upregulated glycolysis rapidly consumes NADH for converting pyruvate into lactate, thereby suppressing mitochondrial respiration. It is noteworthy that the NADH-dependent redox state also regulates the activity of PKB/Akt. Accumulation of NADH due to its suppressed consumption by mitochondria causes inactivation of tumor suppressor gene PTEN protein, a negative regulator of PKB/Akt. Hence, PKB/Akt becomes activated and induces expression of the glycolytic enzymes [262]. This redox-dependent mechanism can be amplified by overexpression of PKB/Akt, a characteristic feature of many tumors [262] (Figure 6). Finally, suppression of OXPHOS and upregulation of glycolysis in a course of tumor progression can be stimulated by changes in cytoplasmic adenine nucleotides, as energy stress due to insufficient mitochondrial ATP synthesis activates the AMP-kinase through increased AMP in the cytoplasm, thus stimulating biosynthesis of glycolytic enzymes in cancer cells [242].

#### 3.4.2. Mechanisms of anti-apoptosis in cancer cells

As already was mentioned, mitochondria play a central role in triggering and establishing apoptotic cell death [263]. The importance of mitochondria is explicitly indicated by the following facts. (i) Stimulation of mitochondrial respiration and ROS generation are early events of apoptosis [264 and references therein]. (ii) Activation and oligomerization of proapoptotic Bax and Bak proteins is markedly suppressed after inhibiton of OXPHOS by oligomycin or antimycin A [263]. (iii) Cybrid osteosarcoma cells lacking respiratory chain are unable to undergo apoptosis [265]. Oncogene p53 is likely one of the key factors in the pathways linking cytotoxic stress to mitochondria-dependent apoptosis. p53 activates the transcription of proapoptotic proteins including Bax, Noxa, Puma, and a p53-regulated apoptosis inducing protein-1, but represses the antiapoptotic genes, such as survivin and Bcl-2 [266–271]. A fraction of p53 translocates into mitochondria [272,273], and this process is followed by hyperpolarization-depolarization transient of the inner membrane, increased ROS production, cytochrome c release and caspase activation [274–278]. According to Zhao *et al*. [272], p53, after having entered the mitochondrion, interacts with MnSOD, thereby decreasing its activity and evoking ROS generation. p53 also binds to BAK and induces its oligomerization, thereby causing permeabilization of the outer mitochondrial membrane and release of cytochrome c, these changes triggering apoptosis [273].

These mitochondria-dependent pro-apoptotic mechanisms appear to be attenuated or even switched off in cancer cells. Obviously, mutations in p53 can be causal for anti-apoptosis. Other reasons for that might be related to specific impairments of mitochondria. In cancer cells, the mitochondria are characterized by defective respiratory chain complexes I and III and decreased β-F_1_-ATPase [279–291], and the type of mitochondrial impairment appears to determine the clinical phenotype [279,290]. Accordingly, benign oncocytomas are characterized by impaired complex I, but enhanced expression of other respiratory chain complexes and matrix enzymes, together with upregulation of mitochondrial tissue content, the latter changes likely compensating the insufficient complex I. In contrast, malignant renal tumors exhibit downregulation of all respiratory chain complexes and β-F_1_-ATPase, in correlation with increased tumor aggressiveness and avoidance of apoptosis [279,290]. The second line of discrimination between the cancer cell types goes along their capacity to produce ROS: whereas many types of cancer cells exhibit excess ROS production [239,240,292–297], some cancer forms show very low ROS levels, together with attenuated apoptosis (reviewed by Lu in 2007 [298]). It is known that generation of ROS in mitochondria steeply increases with build-up of transmembrane potential, ΔΨ [299]. In this regard, Santamaria *et al.* showed recently that oligomycin, an inhibitor of β-F_1_-ATPase, strongly delayed the stauroporin-induced cell death in liver and hepatoma cell lines; it was concluded that β-F_1_-ATPase is required to hyperpolarize mitochondria in order to produce ROS for induction of apoptosis [300]. At the same time, it became known that in colon and renal cancers βF_1_-ATPase is downregulated and the cellular content of mitochondria decreased [279,290]. It was therefore proposed that the cancer cells characterized by reduced activity of β-F_1_-ATPase and low content of mitochondria are unable to produce mitochondrial ROS in amounts sufficient to induce PTP and apoptosis. This property may represent an adaptive strategy of cancer cells to avoid ROS-mediated cell death that contributes to their increased aggressiveness and chemotherapeutic resistance [279,290].

In fact, the cancer cells possess a variety of other means for suppressing the mitochondrial ROS. In breast cancer cells, estrogen, by binding to its mitochondrial receptors, upregulates mitochondrial MnSOD that in turn slows down mitochondrial ROS production and apoptosis [294]. Colon cancers exhibit increased UCP2 expression [301,302], which, through lowering intracellular ROS levels, confers reduced susceptibility to oxidative damage, apoptosis and drug-resistance [303]. In an attempt to reveal the underlying molecular mechanisms, Derdak *et al*. overexpressed UCP2 in human colon cancer cells and showed that it was accompanied by reduced ΔΨ and ROS production and increased oxygen consumption, these changes being associated with inactivation of tumor suppressor p53 through its NH_2_-terminal phosphorylation and induction of the glycolytic phenotype [304]. Notably, realization of the Warburg effect is also linked to promotion of anti-apoptotic and pro-survival mechanisms. Activation of PKB/Akt-dependent signaling through altered redox state and HIF-1α- and IGF-1,2-mediated pathways strongly hinders the apoptotic cell death (Figure 6), as activated PKB/Akt suppresses expression of death genes (Bax, Bak, Smac/Diablo, Fas, Bim, and IGFBP-1), but upregulates antiapoptotic (Bcl-2, Bcl-x_L_, survivin, XIAP) and proliferation-supporting genes (clAP1, clAP2), probably through activaton of NF-kB and CREB [214,236,305–311]. Because the mutated p53 can not effectively counterbalance this mechanism (see above), the PKB/Akt-mediated effect may take over in cancer cells. Importantly, the anti-apoptotic influence of PKB/Akt can be enhanced through another mechanism – functional coupling between the OXPHOS and glycolysis – which is also controlled by this kinase and observed in several types of transformed cells, e.g. breast and liver cancer cells. These cells overexpress hexokinase (HK) type II [312–315] under stimulation by HIF-1α or c-MYC [207]. HK II effectively binds to the mitochondrial VDAC and this process is activated by protein kinase B or Akt (PKB/Akt) [314,316,317], which blocks the activity of glycogen synthase kinase 3β (GSK3β), an inhibitor of HK binding to VDAC [318]. Interaction of glycolysis with OXPHOS supports cancer growth and protects against apoptotic death by multiple means (Figure 6). Due to forming of the HK II-VDAC complex, ATP synthesized in mitochondria is transported via ANT and porin channels to active sites of HK II and used as a preferable substrate for glucose phosphorylation, whereas ADP, another product of HK reaction is returned into the matrix for ATP synthesis. Thus, coupling of glycolysis to OXPHOS enables to amplify the glycolytic flux by increasing the efficacy of substrate supply and removal of product inhibition [319]. In parallel, HK II binding to VDAC stabilizes the mitochondrial outer membrane, thereby suppressing the release of intermembrane proapoptotic proteins and/or blocking association of exogenous proapoptotic proteins (Bax) with the MOM [317]. It has been proposed that association of HK II with VDAC increases the ATP/ADP turnover that utilizes ΔΨ, thereby suppressing the ΔΨ-dependent ROS production in the respiratory chain [76], which underlies the downregulation of mitochondrial ROS production. As a proof for importance of these mechanisms, inhibition of binding of HK II by 3-bromopyruvate or its detachment from mitochondria could be shown to suppress significantly cellular growth and induced apoptosis via mitochondrial signaling cascades [315,320].

Increased glycolysis advances proliferative growth of cancer cells by several ways other than through improving the availability of ATP. For example, a resultant acidity prepares surrounding tissues for invasion, probably by suppressing immune response [319], protects mitochondria from PT pore opening, and inhibits activation of Bax and Bak, thus favoring antiapoptosis in these cells [263]. High rate of glycolysis activates the pentose phosphate pathway that provides the precursors (G-6-P) for biosynthetic processes [321]. Given that stimulated pentose pathway leads to increased NADPH and high levels of reduced glutathione, it also ends up with lower cellular ROS accumulation, thus supporting survival of the cancer cells. Moreover, the inflammatory mediators (e.g. cytokines, ROS, and NO) suppress apoptosis by causing mutations in Bcl2 and p53 proteins [214] or nitration of caspase 9 [322], whereas HIF-1α supports invasion, migration and tolerance to hypoxia by inducing vascular growth and erythropoietin synthesis [323]. It is also known that in a variety of neoplastic cells expression of peripheral benzodiazepine receptor (PBR), a mitochondrial protein associated with VDAC protein, is strongly upregulated [324]. As the PBR exerts a strong protective effect against ROS damage [325], it supports cancer cell survival, despite increased ROS loading.

In the light of these data, different pharmacological means for stimulating apoptosis in cancer cells are under investigation [326]. For example, it has been found that in many types of cancers an appropriate chemo- and radiotherapy can recover the ability of mitochondria to release cytochrome c and activate apoptosis [327–330].

#### 3.4.3. Development of gastric cancer: possible role of altered mitochondrial function?

The biological model of gastric carcinogenesis can be displayed as an inflammation-atrophy-metaplasia-dysplasia-carcinoma sequence [331] that is based on three different intermingled processes. Firstly, chronic active inflammation caused by *H. pylori* creates the background for geno- and phenotypic alterations. Secondly, disruption of the balance between apoptosis and cell proliferation results in mucosal atrophy. Thirdly, progressive loss of differentiation favors establishment of intestinal metaplasia characterized by replacement of intestine-type glands for normal glands [332].

To address the role of mitochondria in gastric disease, we have recently characterized the function of OXPHOS in the biopsies of gastric mucosa taken from the patients suffering from chronic active inflammation [333]. We found that compared to non-active gastritis, the active chronic gastritis (confirmed on the basis of the prominent mononuclear infiltration) was associated with decreased complex I dependent ADP-stimulated respiration rate in the corpus mucosa, whereas this parameter was augmented in the antrum mucosa. Increased OXPHOS in the antrum mucosa was unexpected in light of the evidence that *H. pylori* induces stronger oxidative stress in the antrum than in the corpus [334–336]. However, the inverse changes in OXPHOS in antrum and corpus mucosa could arise from differential effects of *H. pylori* on the balance between the antiapoptotic and apoptotic pathways in distinct parts of the stomach. Indeed, *H. pylori* stimulates apoptosis by triggering cytochrome c release [191,337] and translocation of proapoptotic Bax [192,337] or/and the amino-terminal fragment of bacterial cytotoxin VacA into mitochondria [191]. Based on these data and our observation that mitochondria exhibited normal coupling, the suppressed respiration in the gastric corpus mucosa [333] suggests decreased tissue content of mitochondria due to apoptotic loss of mitochondria and cells. However, *H. pylori* can also impel the cells to slowdown the apoptotic processes, through activation of the cellular inhibitor of apoptosis gene 2 [338] and upregulation of cyclooxygenase-2 (COX-2) [335,339,340]. The products of COX-2 (15d-PGJ2 and PGA1) directly inhibit NF-κB-mediated apoptotic pathways via activating the PPARγ [341,342], whereas PPARγ then accelerates biosynthesis of mitochondria, thus increasing the tissue’s oxidative capacity [343,344]. Given that *H. pylori* upregulates COX-2 in the antral mucosa to a greater extent than in the corpus mucosa [333], activation of PPARγ is expected to be more pronounced in the antral mucosa, which would explain the increased OXPHOS in this region observed by us.

It is generally accepted that transition from chronic gastric inflammation into atrophic gastritis heralds high risk for gastric adenocarcinoma [331,345,346]. The subcellular mechanisms of that transition may involve the mitochondrial dysfunction, as electron microscopy revealed decreased content of mitochondria and increased fraction of abnormal or damaged mitochondria in mucosa with chronic gastritis [347]. In line with this observation, we found that gastric corpus mucosa of patients with pernicious anemia, which is an end-stage condition of corpus dominant atrophic gastritis, exhibits decreased respiratory capacity compared to non-atrophic mucosa [348]. In addition, mitochondria of the atrophic mucosa showed a deficient respiratory complex I and increased coupling of succinate oxidation to phosphorylation [348]. Thus, our data show that, like it occurs in many other diseases, the gastric mucosal atrophy results in remodeling of the systems of OXPHOS, with specific impairment at the level of complex I of the respiratory chain (see Sections 3.1.1 and 3.1.2), but improved function of more distal complexes that may represent an adaptive response.

As already discussed above, impaired function of the complex I of the respiratory chain can be related to mutations of mtDNA and excess production of mitochondrial ROS that oxidizes the mitochondrial proteins and redox centers. It may also rely on alterations of Ca^2+^-dependent effects on complex I function, as in the case of pathologically altered mitochondria in brain (see Sections 2.1 and 3.1.1.). However, this possibility remains to be proven in further studies on gastric mucosa. Nevertheless, irrespectively of the reasons for suppressed function of the complex I in mitochondria of corpus mucosa, the major question regards to its pathophysiological role in inflammation, atrophy and carcinogenesis. In this regard, it is interesting that in early stages of carcinogenesis, even before histological evidence of angiogenesis or invasion, the tumor cells in different tissues exhibit overexpression of HIF-1α [349]. In line with these data, analysis of the gastric biopsies of normal mucosa, *H. pylori*-associated gastritis, intestinal metaplasia, dysplasia, and intestinal and diffuse adenocarcinoma revealed progressively increasing expression of HIF-1α and HIF-2α along with this gastric carcinogenic sequence, and changes in HIF isoforms were accompanied by increased expression of erythropoetin, GLUT-1 and VEGF, all of which represent the targets of HIF [350,351]. Others have found that inhibition of HIF-1α in human gastric cancer TMK-1 cells markedly retarded the tumor growth, angiogenesis, and vessel maturation [352]. Altogether, these data strongly infer that transition from gastric inflammation to cancer is mediated by increased activity of HIF. It is possible that upregulation of HIF isoforms occurs secondarily, in response to altered activities of the upstream PI-3K/Akt/mTOR signaling that promotes growth of tumors [353,354]. This possibility is confirmed by observations that the mammalian target of rapamycin (mTOR), an upstream regulator of HIF, is activated in human gastric cancer, whereas attenuation of PI-3K/Akt/mTOR-mediated signalling by rapamycin effectively blocks HIF-1α in gastric cell lines [355]. Suppressed complex I registered in atrophic gastric mucosa by us [348] may support upregulation of HIF-1α through altered metabolism. (i) ROS, increasingly produced due to impaired complex I, can inhibit the PHDs that should lead to higher activity of HIF-1α (see Section 3.3). (ii) Due to inefficient complex I, utilization of NADH-dependent substrates, like pyruvate, may be hindered, which leads to accumulation of these substrates in the cytoplasm, thereby also stabilizing the PHDs [251] and enabling increases in active HIF-1α. Subsequently, high level of HIF-1α should promote Warburg effect along with suppression of apoptosis, all these changes conferring malignancy to gastric mucosal cells (see Section 3.4, Figure 6). In support of this scenario, the recent data show that human gastric cancer is associated with marked increase in expression of lactate dehydrogenase-5 (LDH-5), in correlation with HIF-1α, VEGF and COX-2. As the overexpression of LDH-5 was found to be more prevalent in advanced tumors exhibiting vessel invasion, and since the patients with lower expression of that enzyme showed higher survival rates compared to those with low levels of expression, expression of LDH-5 was considered to be a useful prognostic marker [356]. Interestingly, we found that mitochondria in atrophic gastric mucosa exhibited improved respiratory control by ADP in the presence of succinate [348]. This suggests that in intact cells, the energetic limitations due to downregulation of OXPHOS and insufficient complex I can be overcome by shifting the preferable substrate for oxidation from pyruvate to succinate. Taken together, it appears that suppressed OXPHOS and deficit in complex I of the respiratory chain may be important factors supporting transition from chronic gastritis to gastric cancer through promoting Warburg effect. Oxygraphic measurement of complex I to complex II activity ratios may therefore serve as a valuable diagnostic tool for detecting the metabolic shift in gastric mucosal cells. Its potential prognostic value requires further experimental and clinical evaluation.

## 4. Conclusions

R. Luft has classified his discovery of the first patient with a mitochondrial dysfunction as a starting point of a “revolution in mitochondrial medicine” [357]. By now the “revolution” has entered a new phase, with a necessity to consider that dysfunction of mitochondria underlies almost all diseases and pathological processes. Indeed, four large groups of diseases with mitochondrial impairments can be distinguished: 1) primary mitochondrial diseases caused by hereditary or acquired mutations within the mitochondrial genome, 2) secondary mitochondrial diseases which are provoked by mutations in the non-mitochondrial genome leading to mitochondriotoxic effects by mutated proteins or crucially altered regulatory processes, 3) acute mitochondrial insults due to ischemia, inflammation, and intoxications, and 4) changes of mitochondrial regulation and function in cancer cells. In any case, impaired mitochondrial function leads to cellular energetic depression which is characterized by diminished phosphorylation potentials, cytoplasmic and mitochondrial Ca^2+^ overload, and accumulation of ROS and toxic proteins.

The central role of mitochondrial dysfunction in cellular pathophysiology is not surprising because (i) the energy state controls directly many of the intra- and intercellular signaling systems, e.g. kinase activities, intracellular Ca^2+^ homeostasis, and proliferation, (ii) the pathogenetic mechanisms of ischemia/hypoxia, inflammation, cancer, and other pathological processes converge at the level of mitochondrial impairment, and (iii) concentration of cellular ATP determines the type of the cell death. Thus, under conditions of limited but persisting mitochondrial functionality, cytochrome c release through a leaky outer mitochondrial membrane but still effective ATP generation sufficient to support the ATP-dependent apoptotic machinery, mitochondria are able to initiate and execute the program of apoptotic cell death. In contrast, if the mitochondrial function completely vanishes due to irreversible opening of the PT pore, the intracellular ATP levels rapidly decrease to amounts insufficient for maintaining apoptosis and the cell has to switch to necrosis. The latter process initiates negative effects on the, even originally non-diseased, neighboring cells and tissue by inducing inflammation that further worsens the clinical phenotype of the respective disease. Both of cell death modes, apoptosis and necrosis, can be taken as the two sites of the common denominator – a mitochondrial cell death. This type of death governs cell fate in the first three types of diseases listed above. In contrast, in tumor cells (disease group 4 above), the cell death machinery and impaired mitochondrial function can be suppressed and compensated, respectively, (i) by effective anti-apoptotic mechanisms that preserve mitochondrial function at least to some extent and period, and (ii) by upregulation of glycolysis and its functional coupling to OXPHOS, these processes representing the main facets of the Warburg phenotype.

Recognition of the central role of bioenergetic failure in cellular pathophysiology has motivated the scientists to elaborate the therapeutic strategies aimed at salvage of the diseased cells by optimizing their energy balance, with mitochondria as a special target to be protected. Retardation of decay of cellular ATP during unfavorable conditions for cell life, e.g. under oxygen and substrate insufficiency, should be a main goal for such interventions, in order to postpone or avoid the necrotic cell death, but at the same time to support apoptosis and inhibit or even reverse the tumor growth. With these goals set, the inhibitors of PT have been proven to be effective in treatment of various animal models of disease [43,48,49]. Furthermore, recent studies have revealed that antidiabetic agents act via inhibiting the mitochondrial complex I, thereby causing activation of AMP-activated protein kinase followed by downstream metabolic alterations (e.g. stimulation of glycolysis and mitochondrial biogenesis) that improve the cellular energy state in muscle cells and ameliorate the insulin resistance [358,359]. A number of drugs that selectively switch mitochondria from the oxidation of fatty acids to carbohydrate oxidation have been found to be beneficial to protect cardiac muscle cells from ischemia/reperfusion injury [360]. Concerning the metabolic therapy of tumors, the attempts to suppress glycolysis by inhibiting the individual enzymes or via releasing HK from mitochondrial binding sites to reverse the Warburg effect have been rather promising. As a complementary or alternative approach, the drug-induced stimulation of PT pore opening and/or the fine-tuned modulation of pro- and anti-apoptotic signaling cascades is expected to promote apoptotic death of cancer cells [reviewed in 16,361].

Keeping in mind the huge complexity of mitochondria with its more than 1200 different proteins, of which many are unknown in functional terms, and our incomplete knowledge on interaction of mitochondria with other cellular structures and systems, we can optimistically look for novel discoveries and a rapid extension of the frontiers of mitochondrial medicine.

## Figures and Tables

**Table 1.** Hereditary mitochondrial diseases

**Type of mutation of mtDNA**	Large scale deletions: KSS, Pearson’s syndrom, PEO
	Point mutations: MELAS, MERRF, NARP, LHON
**Mutations in nuclear genes controlling the stability of mtDNA**	ANT1, Twinkle, POLGI1, TP, TH2, DGUOK, deoxynucleotide carrier
**Mutations in nuclear genes encoding the respiratory chain proteins**	Complex I, NDUFS1, NDUFS2, NDUSFS4, NDUFS8, NDUFV1
	Complex II, SDHA, SDHB, SDHC, SDHD
	Complex III UQCRB, subunit VII
**Mutations in nuclear genes indirectly involved in repiratory chain**	FRDA1 (Friedreich’s ataxia) gene
	Genes responsible for X-linked deafness ataxia and sideroblastic anaemia
	Genes for hereditary spastic paraplegia
	Genes for X-linked deafness-dystonia syndrome
	Genes for autosomal dominant optic atrophy
	Genes responsible for deficiency of coenzyme Q and cardiolipin

**Table 2.** Mechanisms leading to mitochondrial cell death.

**Suppression of ATP synthesis**	Oxidative stress
	Ca^2+^ overload
	Decreased substrate supply (O_2_, fatty acids, glutamate, pyruvate, etc)
	Decreased cytosolic adenine nucleotide concentrations
	Impaired ADP/ATP transport
	Decreased capacity of OXPHOS due to diminished activities of respiratory chain complexes and matrix enzymes resulting from mutations and inhibitions
	Impaired biogenesis of mitochondria
**Changes leading to or associated with apoptosis**	Leaks in mitochondrial outer membrane, loss of cytochrome c
	Opening of the PT pore
	Decreased resistance against Ca^2+^- and ROS-induced stress

**Table 3.** Influence of mitochondria on the velocity of Ca^2+^ waves in a SR vesicle agarose gel.

**Excitable medium**	**Velocity (μm/s)**
SR vesicles (Control)	39.2 ± 16.2^a^ (n = 22)
SR vesicles + thapsigargin	19.6 ± 4.4^a^ (n = 8)
SR vesicles + RHM	57.9 ± 12.9^b^ (n = 20)
SR vesicles + RHM + antimycin A	40.9 ± 10.1^b^ (n = 20)

Spontaneous Ca^2+^ waves in a SR vesicle agarose gel were assessed by confocal scanning fluorescence microscopy with Fluo-3. SR vesicles were isolated from the m. longissimi dorsi of German landrace pigs. Rat heart mitochondria (RHM) were isolated with standard procedures. SR vesicles were incubated in a solution with the following composition (mM): KCl 100, MgCl_2_ 5, Na_2_-ATP 4, Phosphocreatine 10, EGTA 0.04, PIPES 20; Fluo-3 0.01; pH = 7.2. The concentration of agarose gel was 0.66% (86). For Ca^2+^ stimulation we either used a glass tip or a small stripe of paper soaked with Ca^2+^ solution (200 μM). Under the influence of 10 nM thapsigargin, an inhibitor of the SR Ca^2+^-ATPase (SERCA), the velocity of Ca^2+^ waves decreased signifcantly compared to the controls. If mitochondria were added to SR vesicles (together with 10 mM pyruvate plus 2 mM malate as mitochondrial substrates) significantly faster propagating waves were observed. After inhibition of mitochondrial function with 1 μM antimycin A, an inhibitor of complex III of the respiratory chain, this effect was completely abolished. Further details see [86]. Data as mean ± S.D. a, b indicate significant differences between the marked groups (p < 0.01).
